# Fate Analysis of Adult Hippocampal Progenitors in a Murine Model of Fetal Alcohol Spectrum Disorder (FASD)

**DOI:** 10.1371/journal.pone.0073788

**Published:** 2013-09-11

**Authors:** Kenta Kajimoto, Andrea Allan, Lee Anna Cunningham

**Affiliations:** Department of Neurosciences, University of New Mexico School of Medicine, Albuquerque, New Mexico, United States of America; University of South Florida, United States of America

## Abstract

Prenatal alcohol exposure can lead to fetal alcohol spectrum disorder (FASD) and associated behavioral impairments that may be linked to disruptions in adult hippocampal neurogenesis. Social and physical enrichment has been proposed as a potential therapeutic approach toward reversing behavioral deficits associated with FASD and is also a potent stimulator of adult hippocampal neurogenesis. In the present study, we utilized a genetic fate mapping approach in nestin-CreER^T2^/YFP bitransgenic mice to identify the stage-specific impact of prenatal alcohol exposure on the stepwise maturation of adult hippocampal progenitors. Using a limited alcohol access “drinking-in-the-dark” model of FASD, we confirm previous findings that moderate prenatal alcohol exposure has no effect on adult neurogenesis under standard housing conditions, but abolishes the neurogenic response to enriched environment (EE). Furthermore, we demonstrate that this effect is primarily due to failed EE-mediated survival of postmitotic neurons. Finally, we demonstrate that the neurogenic deficit is associated with impaired spatial pattern recognition, as demonstrated by delayed learning of FASD-EE mice in an A–B contextual discrimination task. These results identify a potential maturational stage-specific mechanism(s) underlying impaired neurogenic function in a preclinical model of FASD, and provide a basis for testing regulatory pathways in this model through conditional and inducible manipulation of gene expression in the adult hippocampal progenitor population.

## Introduction

Fetal alcohol spectrum disorder (FASD) encompasses a range of physical, behavioral and cognitive disabilities resulting from prenatal alcohol exposure [1], [2], [3]. Neurological complications in FASD range from severe mental retardation as a consequence of high dose alcohol (fetal alcohol syndrome; FAS) to more subtle behavioral abnormalities as a result of moderate levels of alcohol exposure, including learning deficits, increased anxiety and depression. FASD represents a significant public health problem, with the prevalence of FASD estimated to be as high as 2–5% within the United States and some Western European countries [4] . Despite this, very few empirically supported interventions are available for mitigating the cognitive and behavioral disabilities associated with this spectrum disorder [5].

The production of new neurons in the postnatal and adult hippocampal dentate gyrus is thought to play an important role in learning, memory and mood [6], [7]; and may represent a neural substrate for several behavioral manifestations of clinical FASD [8]. The rate of adult hippocampal neurogenesis has been linked to learning performance, particularly on tasks that require spatial and temporal pattern separation [9], [10], [11]. Potential mechanisms include preferential behavioral activation of newborn dentate granule cells (DGCs) due to their lowered activation threshold and heightened dendritic plasticity [12], [13], and temporal processing as waves of new neurons are added to the hippocampal network [9]. Impaired neurogenesis may also underlie some forms of depression and anxiety [14]. For example, chronic stress reduces neurogenesis and results in depressive-like states in rodent models; whereas chronic treatment with multiple classes of antidepressants increases neurogenesis [14], [15].

Preclinical rodent models of FASD mimic many of the behavioral aspects observed in clinical FASD, including impaired learning, increased anxiety and depression [16], [17], [18]. Furthermore, long-lasting impairments in postnatal hippocampal neurogenesis have been documented following prenatal or early postnatal alcohol exposure (reviewed by [8]). For example, high dose alcohol exposure during the prenatal and early postnatal period results in impaired production and maturation of DGCs in adult rats [19], [20], [21] [22]. More moderate alcohol exposure throughout gestation in mice has no effect on neurogenesis under standard housing conditions, but abolishes the neurogenic response to social and physical enrichment [23]. The mechanism(s) by which alcohol exposure during development leads to enduring neurogenic deficits in adulthood remains unknown. Because each maturational stage of the adult neurogenic lineage (progenitor proliferation, neuronal differentiation and functional integration of postmitotic DGCs) can be differentially regulated by behavioral, environmental and genetic factors, we hypothesized that prenatal alcohol exposure targets a specific maturational step in the adult neurogenic lineage. If so, pinpointing the stage of vulnerability might facilitate therapeutic intervention strategies useful in clinical FASD.

In the present study, we characterized the influence of prenatal alcohol exposure on the stepwise maturation of adult hippocampal progenitors using a genetic fate mapping approach. For these studies, we utilized Nestin-CreER^T2^/YFP mice, which harbor a yellow fluorescent protein (YFP) reporter gene at the Rosa 26 locus and a tamoxifen-inducible Cre recombinase (Cre-ER^T2^) under transcriptional control of the nestin promoter [24]. Tamoxifen administration to nestin-CreER^T2^/YFP mice results in restricted and transient activation of Cre recombinase within nestin+ adult hippocampal progenitors, and induction of YFP reporter expression in all subsequent progeny. This approach facilitates detailed phenotypic fate mapping and distribution analysis of progenitors and their progeny following tamoxifen-induced recombination. Using a limited alcohol access “drinking-in-the-dark” exposure paradigm, we investigated the impact of moderate prenatal alcohol exposure on the adult hippocampal neural progenitor lineage. These studies confirm our previous findings that gestational exposure to moderate levels of alcohol impairs the neurogenic response to enriched environment; and extend those findings to demonstrate impaired survival and integration of postmitotic neurons at late neurogenic stages in FASD mice. We further demonstrate delayed acquisition of contextual discrimination learning in FASD mice, a behavior that is thought to be dependent upon the production of new DGCs. These results shed light on potential stage-specific mechanisms underlying impaired neurogenic responses in preclinical models of moderate FASD, and provide a platform for testing regulatory pathways through conditional and inducible manipulation of gene expression in the adult hippocampal progenitor population.

## Materials and Methods

### Animals

Ethics Statement: Animal experiments were approved by the University of New Mexico Animal Care and Use Committee in accordance with the NIH Guide for the Care and Use of Laboratory Animals. The Nestin-CreER^T2^/YFP bi-transgenic strain used in this study was generously provided by Dr. Amelia Eisch (Department Psychiatry, University of Texas Southwestern Medical Center, Dallas, TX) and previously described [24]. These bi-transgenic mice are on a C57Bl/6J background. All mice were housed in reverse 12-hour dark / 12-hour light cycle (lights off at 08∶00 hours). Food and water were available *ad libitum* except during the maternal drinking period during which water (but not food) was withheld as described below.

### Prenatal Alcohol Exposure

For these studies, we utilized a limited access paradigm of maternal drinking that was previously established for C57Bl/6J mice [16]. To obtain stable drinking levels across mice, 60 day old nestin-CreER^T2^/YFP female mice were first subjected to a ramp-up period in which the normal drinking water was replaced with 0.066% saccharin containing 0% ethanol (2 days), 5% ethanol (2 days) and finally 10% ethanol, for 4 hrs per day from 10∶00–14∶00 (2 weeks prior to pregnancy and throughout gestation). Female mice offered 0.066% saccharin without ethanol during the same time periods and throughout pregnancy served as controls. Following one week of 10% ethanol or saccharin consumption post-ramp, individual females were placed into the cage of singly housed males for 2 hours from 14∶00–16∶00, immediately following the daily drinking period, for five consecutive days. Water and food were available *ad libitum* in the male cages. Females were then returned to their home cages after each 2 hour mating session, where they continued on the limited access schedule of ethanol or saccharin exposure for 4 hours per day throughout gestation. Ethanol and saccharin concentrations were halved every two days beginning on the day of birth, and returned to drinking only water only on day 5. A consumption volume during the 4 hour access period was determined for each mouse from the onset of the drinking paradigm. Offspring were tail-clipped and genotyped at weaning and subjected to enriched or non-enriched living conditions as outlined below. The limited access maternal drinking paradigm has no impact on litter size, offspring weight or maternal care [16].

### Blood Ethanol Measurements

Blood ethanol concentrations were determined using an alcohol dehydrogenase enzymatic method as previously described [16]. Briefly, blood samples obtained from submandibular vein (40 µl) were treated with 2 ml of 3.5% (v/v; 0.58 M) perchloric acid and centrifuged to obtain serum. Forty microliters of serum or ethanol standard (0–400 mg/dl ethanol) were incubated in reaction buffer (10 units alcohol dehydrogenase, 2.0 mM NAD, 0.5 M Tris-HCl, pH 8.8) for 15 minutes at 30°C and optical densities measured at 340 nm using a Beckman DU380 spectrophotometer. Blood ethanol concentrations were calculated from the standard curves using regression analysis.

### Environmental Enrichment (EE)

Mice were gender segregated at weaning. Male pups were used for further study to avoid potential gender-specific effects of prenatal alcohol on hippocampal function and neurogenesis. To activate Cre-mediated recombination, male offspring received daily i.p. injections of tamoxifen (TAM; 180 mg/kg dissolved in 10% EtOH/90% sunflower oil; ∼150 µl per mouse) for five consecutive days beginning at postnatal day 40. TAM-treated mice were then placed into either the standard housing condition (3 mice per 28 cm×18 cm×13 cm mouse cage without running wheels or toys) or were placed in EE housing conditions (6 mice per 48 cm×27 cm×20 cm cage with 2 running wheels, one ladder, one tunnel and multiple hanging toys that were changed weekly). All mice were perfused for histological analysis after 10 weeks of standard or EE housing. To control for potential litter effects, each cage contained individual offspring from 2–3 different litters for each experiment. Twelve litters were used for these studies (6 litters for each alcohol treatment group).

To monitor interaction of Sac and FASD mice with their environment, movement within the EE home cage was recorded and analyzed using the Noldus EthoVision 3.0 video tracking system (Noldus, Leesburg, VA). For this analysis, three target zones encompassing the running wheel, ladder and tunnel were outlined with minimal border zones and the duration of time spent in each environmental zone was recorded over a one hour period. It should be noted that cage-mates were removed from the EE during the recording session (n = 5 mice per treatment group taken from 2–3 different litters per group).

### Histology

Mice were overdosed with sodium pentobarbital (150 mg/kg, i.p.; Fort Dodge Animal health, Fort Dodge, IA), and transcardially perfused with 0.1 M phosphate-buffered saline (PBS) containing 0.1% procaine and 2 U/ml heparin, followed by 4% paraformaldehyde (PFA) in PBS. Brains were removed and post-fixed in 4% PFA overnight, followed by cryoprotection with 30% sucrose in PBS. The right hemisphere of each brain was sectioned at 30 µm thickness in the coronal plane using a freezing sliding knife microtome. Sections were stored at −20°C in cryoprotectant (25% glycerol, 25% ethylene glycol and 50% of 0.1 M phosphate buffer) until used for immunostaining.

Slide mounted and free floating-sections were subjected to immunofluorescence staining protocols as previously described [25], [26]. The following antibodies were used: mouse anti-NeuN (1∶1000, Millipore, Billerica, MA), rabbit anti-DCX (1∶500, Cell Signaling Technology, Danvers, MA), mouse anti-PCNA (1∶1000, Santa Cruz Biotechnology, Santa Cruz, CA), mouse anti-GFAP (1∶1000, Sigma-Aldrich, St. Louis, MO ), chicken anti-GFP (1∶1000, Invitrogen, Carlsbad, CA), and rabbit anti S-100β (1∶1000, DAKO, Carpunteria, CA). Sections were subsequently incubated with donkey secondary antibodies directed against mouse, rabbit or chicken IgG. Secondary antibodies were conjugated to Cy3, Cy5, FITC (1∶250) or biotin (1∶1500) (Jackson Immunoresearch, West Grove, CA). Preincubation with 10 mM of sodium citrate (pH 6.4, 80°C) for 30 minutes was required for antigen retrieval when staining for PCNA. Briefly, sections were rinsed with 0.1% of Tween-20 in PBS (PBS-T) for 10 minutes, permeabilized with 0.4% of triton X-100 in PBS for 20 minutes, followed by incubation in blocking solution (0.1% BSA and 10% normal donkey serum in PBS-T) for 1 hour. Sections were then incubated overnight with primary antibodies in 0.5% BSA/PBS-T at the indicated dilutions. YFP immunofluorescence was visualized using biotinylated secondary antibody and the Tyramide-Plus signal amplification kid (PerkinElmer Life Science, Boston, MA). Sections were counterstained using DAPI nuclear dye and coverslipped using Prolong® Gold Antifade reagent (Invitrogen). All images were taken using an Olympus DSU spinning disk confocal microscope.

### Stereology

The number of YFP+ neurons was estimated within the right dentate gyrus using the optical fractionator method and Stereoinvestigator^TM^ software (Microblightfield, Williston, VT) linked to an Olympus DSU spinning disk confocal microscope. The contour of the dentate gyrus was manually outlined in each section using a 20×objective. YFP+ cells were counted in every 8^th^ section (240 µm apart) through the rostro-caudal extent of the right dentate gyrus. For immunophenotyping the YFP+ cells, 100–200 YFP+ cells were sampled and scored for each phenotypic maker using rapid z-analysis on the confocal microscope. Based on co-localization for the following markers, YFP+ cells were categorized as type-1 stem cells (GFAP^+^/S100β^−^) transient amplifying progenitor cells (PCNA^+^/DCX^−^), proliferating neuroblasts (PCNA^−^/DCX^+^), postmitotic neurons (NeuN^+^), or astrocytes (GFAP^+^/S100β^+^). The number of cells within each phenotypic category was calculated by multiplying the percentage of cells in each category by the total number of YFP^+^ cells as estimated by stereology.

### Contextual Fear Discrimination Learning

Male C57Bl/6J Saccharin controls and FASD mice were assessed for contextual fear discrimination learning. After 4 weeks exposure to EE housing, mice were trained five consecutive days in the A–B contextual fear discrimination learning task, as modified from Sahay et al., [27]. Each mouse was placed into either Context A (foot shock) or Context B (no foot shock) for 90 s, followed by applied foot shock (0.8 mA) to only Context A for 2 s. Following a second 90 s interval, another 1 s foot shock was applied only in Context A. Three hours after the first session, mice were exposed to a second identical training session, except that the mouse initially subjected to Context A with foot shock was now subjected to Context B without footshock. Context A was a standard chamber with a stainless steel floor, clear plexiglas front wall, and aluminum side and back walls. Context B was a similar chamber, except the floor was a wire mesh non-shock floor and the side and back walls were covered in striped black and white contact paper. Mice were transported into a holding room located just outside of the context discrimination room for at least one hour before testing. The context chambers were cleaned with 70% isopropyl alcohol between each session. The order of testing in Context A *vs.* B was altered every day for each mouse. Trials were recorded using a digital camera and mice were scored for time spent freezing during the first 90 s of each trial (observer blinded to treatment). A mean discrimination score was calculated as: (freezing score context A – freezing score context B)/(freezing score context A + freezing score context B). Thus, higher discrimination scores indicate better contextual discrimination.

### Statistical Analysis

Data were analyzed by ANOVA with appropriate post hoc analysis using Prism or SPSS (v.20) software programs. Data are expressed as means ± S.E.M., with p<.05 considered statistically significant.

## Results

### Drinking behavior and BECs in Nestin-CreER^T2^/YFP Mice

**Figure 1:**
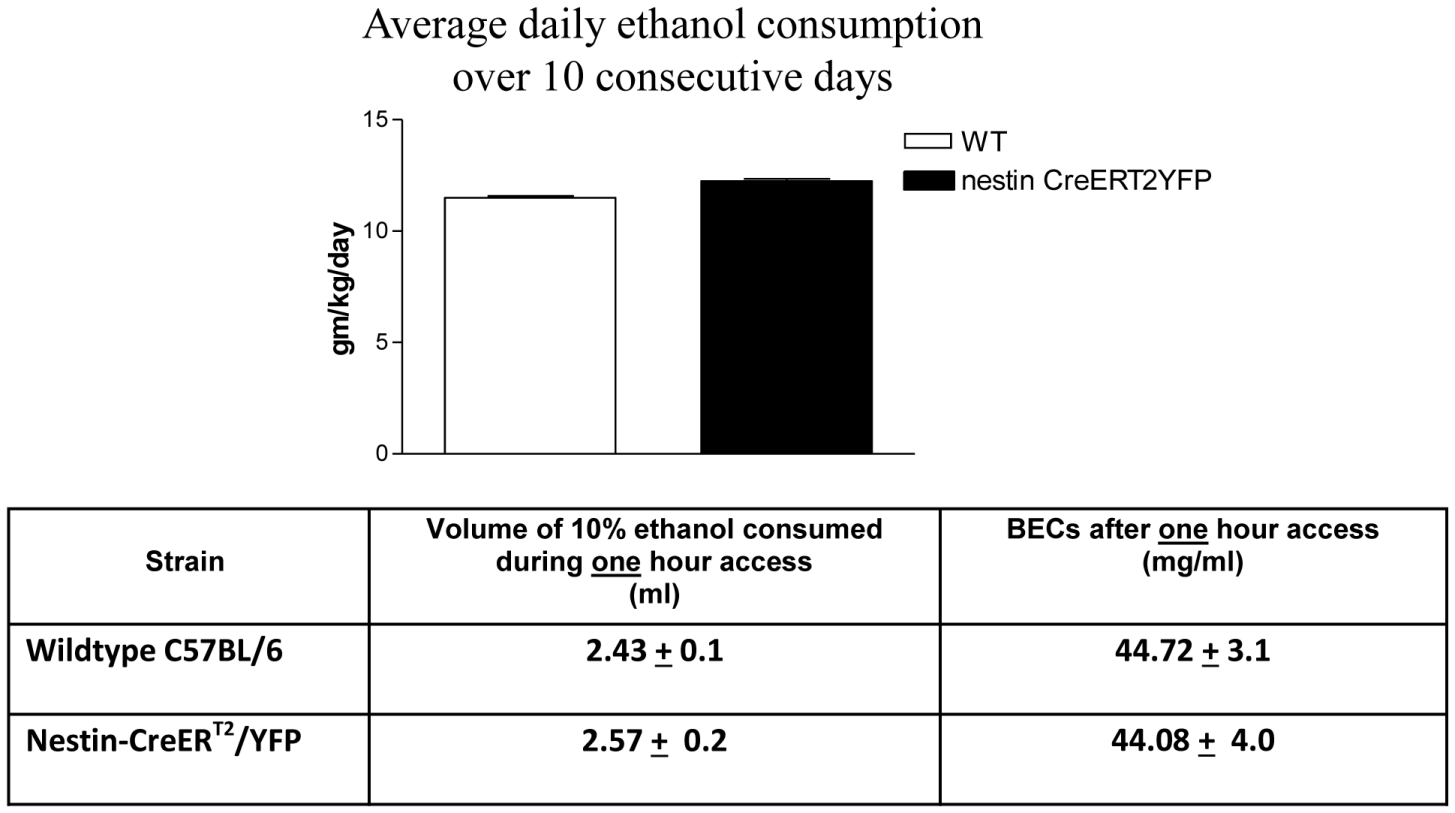
Alcohol Consumption and BECs in Nestin-CreER^T2^/ YFP mice. (A) Average daily consumption of ethanol (mg/kg/day) over 10 consecutive days in wild type (WT) and transgenic mice (n = 5 mice/group) (B) Alcohol consumption (ml) and BEC (mg/dl) after only one hour alcohol access (n = 5 mice/group). Data expressed as means ± S.E.M.

The limited access model of moderate prenatal alcohol exposure used in the current study to generate FASD offspring was previously described [16]. Briefly, female mice were offered 10% ethanol in 0.066% saccharin for four hours during their dark cycle (10∶00–14∶00), beginning one week before pregnancy and continuing throughout gestation. This drinking paradigm results in an average blood ethanol concentration (BEC) of 68 mg/ dl and 88 mg/dl after 2 and 4 hours of drinking, respectively; with ethanol consumption rate predictive of BECs [16]. As shown in Figure 1, the average alcohol consumption rate and BEC values (as assessed after one hour access) were not different comparing wildtype (WT) and transgenic mice, indicating that neither drinking behavior nor alcohol metabolism were altered in the nestin-CreER^T2^/YFP mice compared to background strain.

### FASD Mice Display Impaired Enrichment-mediated Neurogenesis

**Figure 2:**
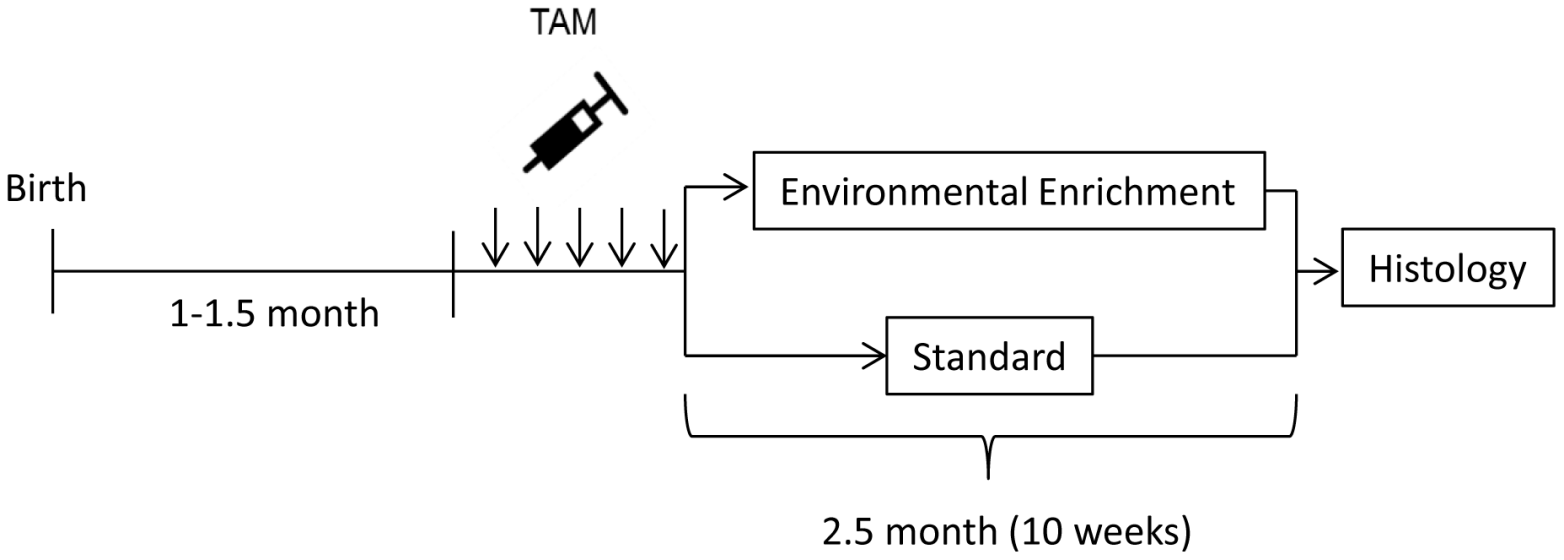
Experimental Design. Tamoxifen (180 mg/kg, i.p.) was administered once each day for 5 consecutive days to induce YFP expression in adult hippocampal progenitors of 4–6 week old nestin-CreER^T2^/YFP mice. Tamoxifen-treated mice were exposed to standard or enriched housing conditions for 10 consecutive weeks prior to histological analysis.

To evaluate the impact of FASD on hippocampal neurogenesis in adulthood, we estimated the number of YFP+ cells in Sac nestin-CreER^T2^/YFP *vs.* FASD nestin-CreER^T2^/YFP mice housed under standard or EE living conditions for 10 weeks. YFP reporter expression was induced in nestin^+^ NSCs by tamoxifen (TAM) administration to all mice for 5 consecutive days (180 mg/kg i.p.) prior to placement into standard or EE housing for 10 consecutive weeks (Figure 2). Therefore, all mice were approximately 3.5 months of age at the time of sacrifice. Previous studies have demonstrated that the TAM dosing regimen used here is optimal for phenotypic fate mapping, resulting in a gradual buildup of YFP^+^ cells as adult hippocampal progenitors proliferate and give rise to postmitotic DGCs, reaching a steady state by approximately 30 days post TAM treatment [24].

**Figure 3:**
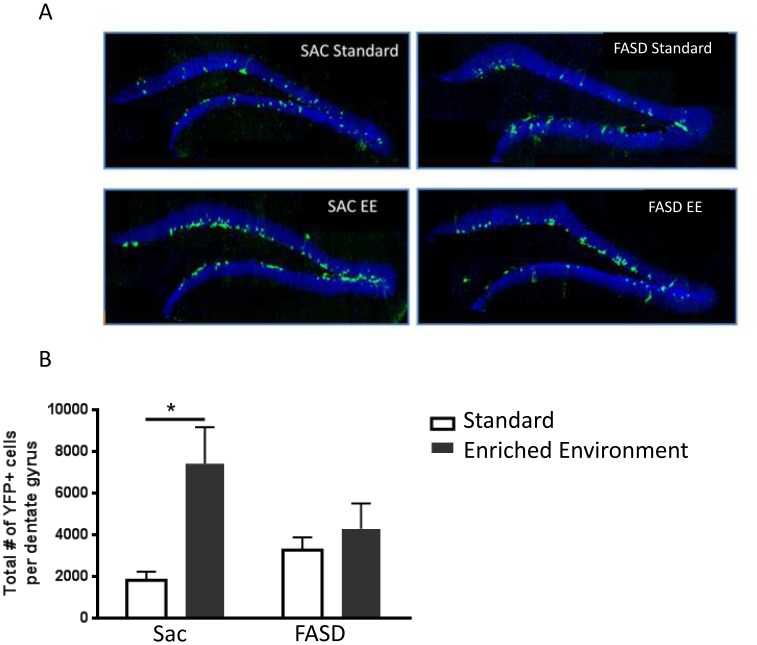
The neurogenic response to EE is blunted in FASD mice. (A) Confocal images of hippocampal dentate: YFP immunofluorescence (green) and DAPI nuclear stain (blue). (B) Total number of YFP positive cell in dentate gyrus 10 weeks after TAM injection. Post hoc analysis revealed a significant increase in the total number YFP+ cells in Sac nestin-CreER^T2^/YFP mice, but not FASD nestin-CreER^T2^:YFP mice housed under environmental enrichment (*p<0.05, n = 8 mice per group).

As shown in Figure 3, the mean number of total YFP^+^ cells was increased approximately 4-fold by EE compared to standard housing in Sac nestin-CreER^T2^/YFP mice (1,821±406 *vs.* 7,424±1,737 YFP+ cells under Standard housing *vs.* EE, respectively). In contrast, there was no effect of housing on the number of YFP^+^ cells in FASD nestin-CreER^T2^/YFP mice (3,261±617 *vs.* 4,291±1,217 YFP+ cells under Standard housing *vs.* EE, respectively). Two-way ANOVA revealed a significant housing effect, (F (1, 28) = 8.72, p = .006), and significant FASD × housing interaction, (F (1, 28) = 4.15, p = .05). *Post hoc* analysis confirmed increased YFP^+^ cell number under conditions of EE in SAC nestin-CreER^T2^/YFP mice, but not in FASD nestin-CreER^T2^/YFP mice. There was not a significant difference in the total number of YFP-labeled cells in Sac vs. FASD nestin-CreER^T2^/YFP mice under standard housing, even though labeling appeared to be slightly higher in FASD mice.

### FASD Abolishes the Beneficial Effects of EE on the Survival of Postmitotic Adult-generated DGCs

To determine the phenotypic distribution of YFP^+^ cells across the neurogenic lineage, we surveyed the YFP^+^ cell population in Sac and FASD nestin-CreER^T2^/YFP mice for co-expression of markers specific for each maturational stage. YFP^+^ cells were categorized as radial-glia-like type-1 NSCs, transit amplifying progenitor cells (TAPs), neuroblasts, postmitotic immature neurons, mature neurons or astrocytes based on expression of immunohistochemical markers as indicated in Table 1 [28], [29], [30].

**Table 1:** Phenotypic markers used to identify maturational stages of YFP+ cells include GFAP (glial fibrillary acidic protein), S100β (a calcium binding protein expressed in mature astrocytes), PCNA (proliferating cell nuclear antigen), DCX (doublecortin; a microtubule-associated protein expressed by neuroblasts and immature neurons) and NeuN (neuronal nuclear antigen).

Cell Type	Phenotypic Markers
**Type I NSC**	GFAP ^+^/S100β^−^
**TAP**	PCNA^+^/DCX^−^
**Neuroblast**	PCNA^+^/DCX^+^
**Immature Neuron**	PCNA^−^/DCX^+^
**Neuron**	DCX^−^/ NeuN^+^
**Astrocyte**	GFAP^+^/S100β^+^

**Figure 4:**
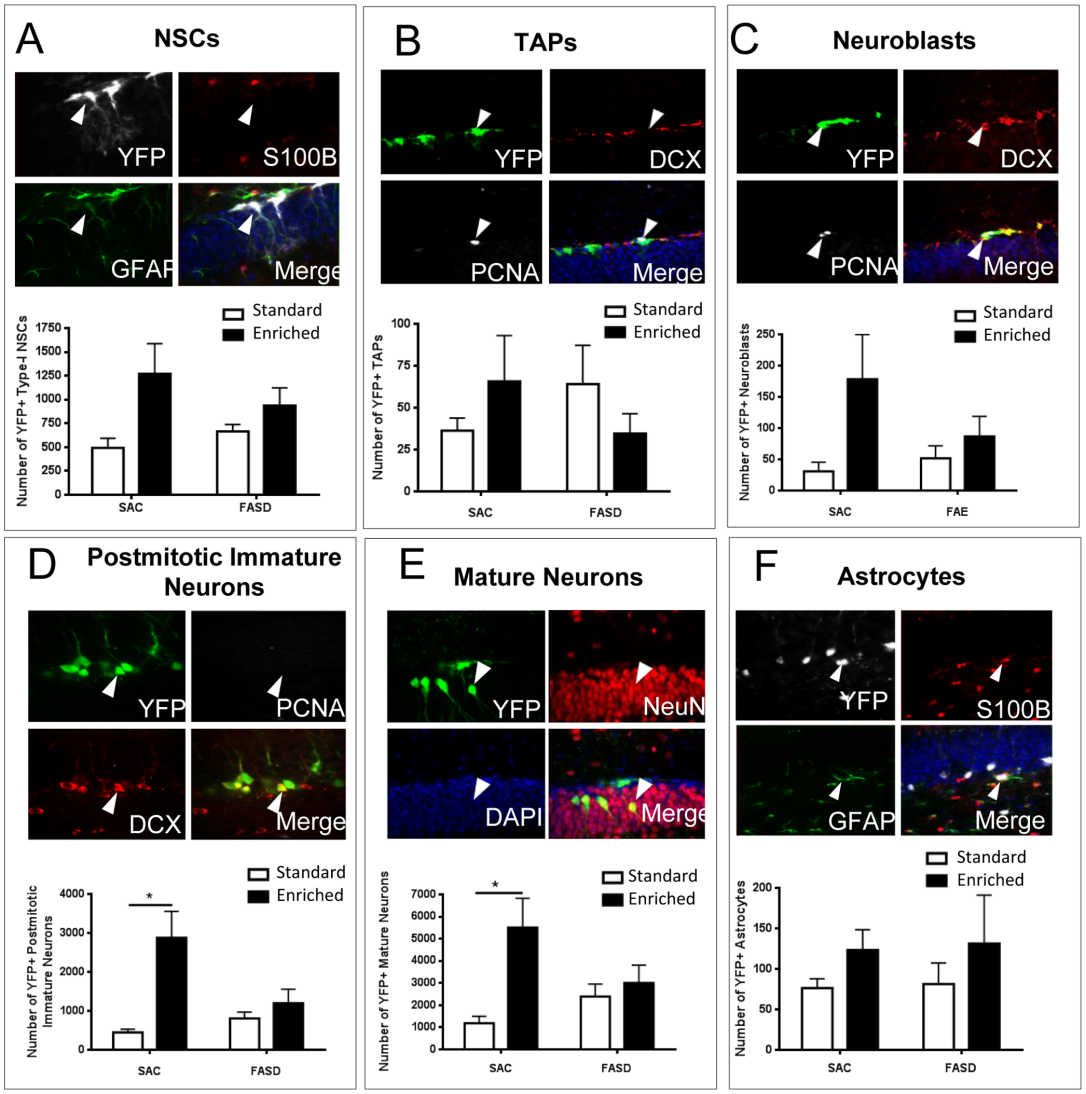
Fate analysis of YFP+ cells in Sac and FASD mice exposed to EE and standard housing conditions. For each panel, representative confocal images are shown at top and quantitative data at bottom(A) Type-1 stem cells were identified as GFAP+ (green) and S100beta-(red), (B) Transiently amplifying progenitor cells (TAPs) were PCNA positive (white) and DCX negative (C) Neuroblasts were PCNA+ (white) and DCX+, (D) Postmitotic immature neurons were DCX+ (red) and PCNA- (white), (E) Mature neurons were NeuN positive (red),(F) Astrocytes were GFAP+ (green) and S100beta+ (red). Data represent means ± SEM, n = 8 mice per group. *p<0.05 post-hoc analysis.

As shown in Figure 4, YFP^+^ cells represented the entire neurogenic lineage in all treatment groups. Two-way ANOVA revealed a significant main effect of housing on YFP^+^ type-1 stem cells (F (1, 28) = 7.34; p = .011) and neuroblasts (F (1, 28) = 4.94; p = .035), but FASD × housing interaction was not significant for these cell types. However, FASD significantly impaired EE-mediated increases in the number of postmitotic immature neurons (F (1, 28) = 12.69; FASD × housing interaction, p = .001) and mature neurons (F (1, 28) = 8.69; FASD × housing interaction, p = .006). There was no significant effect of FASD or housing on the number of YFP+ astrocytes, indicating that abolishment of EE-mediated neurogenesis in FASD mice was not due to a progenitor switch towards astrocyte lineage. No significant effects of FASD or housing were observed for the number of YFP+ TAPs.

**Figure 5:**
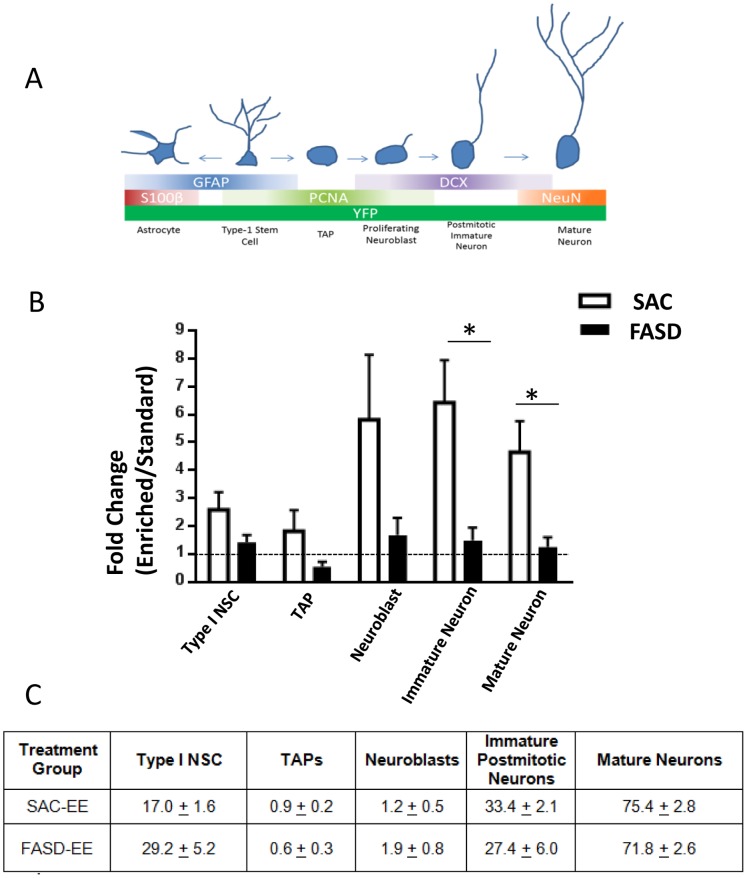
Fold Change and Distribution of YFP+ Cells in Sac-EE and FASD-EE. (A) Depiction of markers used to identify neurogenic cell types. (B) Number of YFP+ cells in EE normalized to Standard housing within each alcohol treatment group. Data are expressed at the mean normalized values + S.E.M. (n = 8 mice per group, *p<0.05 unpaired t-test) (C) Percent distribution of cell types among all YFP+ cells in SAC-EE vs. FASD-EE mice. Data are expressed as the mean percentage of all YFP+ cells in each category ± S.E.M (n = 8 mice per group).

To account for potential differences in the YFP labeling efficiency in Sac vs. FASD nestin-CreER^T2^/YFP mice, data were normalized to standard housing conditions within each alcohol treatment group. Statistical analysis revealed significant differences between Sac vs. FASD groups in the number of immature (p = 0.007) and mature (p = 0.01) postmitotic neurons, but not for type I NSCs, TAPs or neuroblasts under EE conditions when data were normalized to standard housing conditions (Figure 5B). However, the overall proportion of YFP+ cells comprising the various cell types in the neuronal lineage did not display a significant shift, although there was a tendency for a higher percentage of early progenitors amongst all YFP+ cells in FASD-EE mice (Figure 5C). This suggests that fewer postmitotic neurons in FASD-EE mice is not associated with a significant buildup of early progenitors, suggesting that the effects of FASD are not due to stalled differentiation.

### Motion Tracking in Sac and FASD Mice within EE Cage Environment

Recent studies have demonstrated that EE-mediated neurogenesis is correlated with the manner in which mice interact with their environment [31]. To ensure that the impaired EE-mediated neurogenesis in FASD mice was not due to reduced interaction with the environment, we utilized the EthoVision Motion Tracking system to quantify duration spent within cage zones, as described under the methods section. There were no significant differences between Sac-EE and FASD-EE mice in the duration of time spent in the various cage zones. Seconds spent in various cage zones during a one hour tracking session for Sac-EE *vs.* FASD-EE mice, respectively, were as follows: running wheel (565±115 *vs.* 466±236), ladder (266±44 *vs.* 119±20), tunnel (248±61 *vs.* 309±35, n = 5 mice per group). This suggests that the resistance to EE-mediated neurogenesis in FASD mice is unlikely due to failure to interact with environment.

### FASD Mice Display Delayed Learning in an A–B Context Discrimination Task

**Figure 6:**
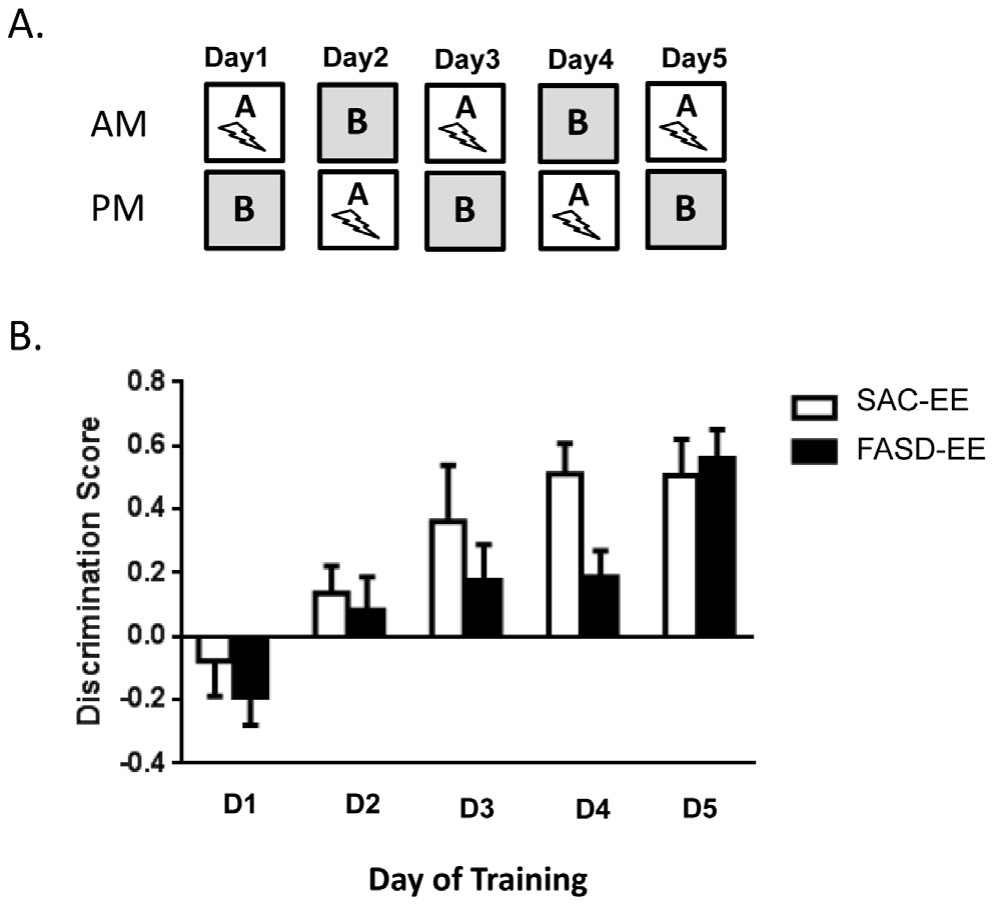
Delayed learning acquisition in an A–B Context Discrimination task in FASD mice. (A) Experimental design of the contextual discrimination task. (B) Daily discrimination scores for Sac-EE and FASD-EE mice over five consecutive days of training. Data are expressed as the means ± S.E.M., n = 5 mice per group.

We next compared Sac-EE and FASD-EE mice for their ability to learn an A–B contextual discrimination task, which requires mice to discriminate between two similar contexts. Performance on this learning task has previously been shown to depend on survival and integration of adult-generated DGCs [27], [32], [33]. The ability to discriminate between similar contexts was assessed by comparing the time spent freezing in the shock context A *vs.* the non-shock similar context B, expressed as the discrimination ratio: (freezing context A – freezing context B)/(freezing context A + freezing context B). As shown in Figure 6, FASD-EE mice required a longer time to learn the task compared to Sac-EE mice. Repeated two-way ANOVA (training day *vs.* treatment) revealed significant main effects of training days (F (4, 64) = 21.22; p < .0001) and alcohol treatment (F (4, 16) = 5.064; p = .0118). This suggests that both control and FASD mice learned to discriminate contexts over the five testing days, but FASD mice displayed a significantly slower rate of learning compared to control mice. *Post hoc* t-test of discrimination scores at each testing day showed that FASD mice displayed significantly lower discrimination scores compared to control mice at testing day 4 (p = .0425); however, the level of discrimination in FASD mice reached the same level as control mice at testing day 5. Thus, Sac-EE mice could discriminate between contexts by training day 4, whereas FASD-EE mice required 5 days of training. These results demonstrate that impaired neurogenesis in FASD-EE mice is reflected by delayed acquisition of a spatial pattern recognition task previously shown to be reliant on the functional integration of newborn DGCs.

## Discussion

In the present study, we utilized nestin-CreER^T2^/ YFP mice to elucidate the stage-specific effects of moderate prenatal alcohol exposure on adult hippocampal neurogenesis. The nestin-CreER^T2^/YFP mice provide the opportunity to survey the full constellation of maturational cell types in the neurogenic lineage, from primitive type I stem cells to mature postmitotic neurons, at a single time point following tamoxifen-induced recombination in a cohort of nestin^+^ cells within the SGZ. Our results confirm previous findings of an impaired neurogenic response to EE in FASD mice [23], and suggest this is due primarily to a failed survival-promoting effect of EE on postmitotic neurons. In addition, we found that FASD-EE mice display delayed learning in a contextual fear conditioning task that is known to rely on adult neurogenesis. Taken together, these findings demonstrate impaired neurogenic function in a preclinical mouse model of FASD and identify postmitotic neurons as the cells most vulnerable to the detrimental effects of moderate prenatal alcohol exposure.

The limited access “drinking in the dark” alcohol exposure paradigm used in the present study is a voluntary consumption paradigm modified from a mouse model originally established by Boehm and colleagues [34]. The limited access to 10% ethanol for 4 hours daily results in average BECs in mouse dams of 88 mg/dl throughout the gestational period [16]. While this is considered a moderate exposure paradigm experimentally, it mimics daily drinking to legal limits of intoxication (80 mg/dl BECs legal intoxication) throughout the first and second trimesters of pregnancy in humans, since the third trimester equivalent of CNS development occurs during the first 2 postnatal weeks in rodents [35]. This paradigm is relevant to human drinking behavior, since most women who drink during pregnancy report greatly decreased alcohol consumption by the third trimester [36]. The limited access mouse model of FASD does not result in altered maternal care, pup weight, litter size, food and water consumption or altered locomotor activity in adulthood[16]. However, these FASD mice exhibit significant deficits in several hippocampal dentate-specific tasks including delay fear conditioning, trace fear conditioning and nonmatching to place radial arm maze [16], and long-lasting deficits in hippocampal NMDA-receptor-dependent long-term potentiation (LTP) in adulthood [37]. Our study further shows that EE-mediated neurogenesis is abolished in these mice, even though their exploratory behavior within the complex environment is not impaired. Using a continuous gestational exposure paradigm in a mixed strain background (BECs ∼121 mg/dl), we previously found no differences in the size of the SGZ progenitor pool during adulthood, but a significant impairment of EE-mediated neurogenesis as assessed by BrdU labeling of progenitors. The current study confirms and extends those findings to include the limited access paradigm and identify late stage neurogenesis to be most vulnerable.

It is important to note that our EE conditions included continuous access to running wheels in addition to increased social and physical environmental complexity. Both voluntary running and environmental complexity stimulate adult hippocampal neurogenesis, but through different mechanisms [38] [39] [40]. Continuous access to a running wheel has transient proliferative effects on early progenitors in mice, peaking within 10 days and returning to baseline by one month [41], whereas environmental complexity in the absence of running primarily enhances the survival and functional integration of newly generated postmitotic neurons [42]. Running and environmental complexity have additive effects on neurogenesis when combined [43]. Although we did not observe a significant FASD × housing interaction on the number of YFP+ early progenitors, the significant housing effect on type I NSCs and neuroblasts is consistent with previous studies [40], [41], [44]. That a significant FASD × housing interaction (p = .001) was detected only for the number of postmitotic neurons suggests that the impaired neurogenic response to EE in FASD mice is largely due to failed EE-mediated survival and integration of newly generated neurons, and not due to an inadequate pool of early progenitors.

Several studies have demonstrated a negative impact of prenatal or early postnatal alcohol exposure on adult hippocampal neurogenesis (reviewed in [8]). Pertinent to our study, Hamilton et al., (2012) reported that rats exposed to high dose (binge) alcohol (∼300 mg/kg BEC) during the third trimester equivalent (postnatal day 4–9), show impaired neurogenesis under standard housing conditions, which could be rescued by 12 days of wheel running followed by exposure to environmental complexity [45]. Similarly, Boehme et al., (2011) reported that alcohol exposure throughout all three trimester equivalents (∼200 mg/dL BEC) resulted in impaired progenitor proliferation in young adult female mice that could also be reversed by voluntary wheel running [22]. In contrast, our FASD mice showed no deficits in baseline neurogenesis under standard housing, but displayed resistance to the beneficial effects of EE. The discrepancy between these studies could be due to species differences, but are more likely due to differences in the dose and timing of alcohol exposure relative to hippocampal development.

Development of the human hippocampus occurs during the second and third trimesters. In rodents, formation of the dentate granule cell layer (GCL) begins during the third week of gestation (second trimester equivalent) and continues postnatally, with the adult SGZ becoming established by the end of the second postnatal week (third trimester equivalent) [46]. In rodents, the oldest DGCs are generated during the late gestational period from progenitors of the subventricular zone that constitute the secondary germinal matrix of the dentate, whereas younger DGCs are generated during postnatal development from a tertiary matrix that gradually gives way to the SGZ by the second postnatal week [47], [48], [49], [50]. Recent studies suggest that the long-lasting progenitors within the adult SGZ originate from a distinct population of neural stem cells within the ventral hippocampus generated during the perinatal period [51]. Synaptic input from the entorhinal cortex begins during late gestational periods in the rodent and continues to reach adult levels by postnatal day 25 in the rat [52]. Clearly, alcohol exposure during late gestation *vs.* early postnatal periods could influence distinct aspects of dentate development that lead to long-lasting deficits in neurogenesis that may manifest with distinct characteristics. In our FASD model, hippocampal progenitors are exposed to alcohol during the generation of the oldest DGCs, suggesting that it may be the neurogenic niche environment that is primarily altered by alcohol rather than the postnatal SGZ progenitors themselves.

Brain-derived neurotrophic factor (BDNF) is one niche factor affected by prenatal alcohol that is known to play a critical role in EE-mediated neurogenesis [53], [54]. EE and voluntary exercise elevate levels of BDNF and its receptor, TrkB, in adult hippocampus [22], [55], [56], [57] and enhance synaptic transmission and neuronal excitability [58]. Furthermore, knockdown of BDNF levels in heterozygous BNDF+/− mouse impairs EE-mediated neurogenesis [54]. BDNF levels in adult hippocampus have been reported to decrease [18], [59] or to remain unchanged [22] following prenatal alcohol exposure. It will be important to determine whether impaired neurogenesis in response to EE in our paradigm is associated with impaired BDNF signaling; if so, it might be possible to utilize conditional and inducible genetic approaches to enhance BDNF signaling in FASD mice in an attempt to restore neurogenic responsiveness to EE.

Another potential mechanism that could underlie impaired neurogenic responses to EE in FASD mice is altered electrophysiological function of the existing hippocampal circuitry. Neurogenesis is tightly linked to neuronal excitation [60], [61]. Activity-dependent synaptic integration into existing hippocampal circuitry is one of the most important factors in determining long-term survival of early postmitotic DGCs. Early 1–3 week old progenitors and neuroblasts require GABAergic and glutamatergic input for survival [62], [63], whereas 4–6 week old postmitotic neurons require activity dependent synaptic integration during a critical period of heightened plasticity and lowered threshold for long-term potentiation (LTP) [12]. Expression of both NR1 [64] and NR2B [32] NMDA receptor subunits in newborn DGCs is important for survival and neurogenesis-dependent LTP, respectively. Thus disruptions of the existing circuitry or impaired expression of NMDA receptor subunits in newly generated DGCs could abolish activity-dependent integration and survival of postmitotic neurons in response to behavioral challenge.

Gestational alcohol is known to have marked effects on the electrophysiological properties of adult dentate. Prenatal alcohol exposure results in decreased dentate LTP [37], [52], [65], [66], decreased expression of both NR1 and NR2B NMDA receptor subunits [67], and decreased NMDAR1 expression [68], [69]. GABA receptor expression and activity is also altered by prenatal alcohol exposure in rodents [70], [71]. It will be important to determine whether these alterations in receptor expression and excitation are restricted to the existing DGC population, or also occur in adult progenitors and their progeny. In the current study, neurogenic deficits in FASD mice were only observed under conditions of behavioral challenge (EE). Given our moderate alcohol exposure paradigm, it is possible that synaptic transmission is sufficient to support basal rates of neurogenesis, but suboptimal and rate-limiting under conditions of behavioral challenge such as EE. A recent study has identified a novel niche mechanism that drives excitation-neurogenesis coupling; regulation of Wnt signaling by local existing DGCs in an activity-dependent manner [72]. This pathway should also be evaluated in the context of FASD.

The impairment of EE-mediated neurogenesis in our FASD model was associated with delayed learning in a context discrimination task that requires mice to distinguish between two similar contexts as a test of pattern separation. Sahay et al., (2011) previously demonstrated that enhancing the survival of newly generated DGCs in adulthood is sufficient to improve performance on this task [27]. Conversely, ablation of neurogenesis by irradiation [33] or by conditional induction of apoptosis in adult progenitors [11] impairs performance on this task, as does inducible deletion of the NR2B NMDA receptor subtype in adult progenitors [32]. Clelland et al., (2009) demonstrated impaired spatial pattern separation as measured in a two-choice touch screen task and a nonmatching to place radial arm maze task following depletion of adult hippocampal progenitors [10]. Saxe et al. (2006) showed that ablation of hippocampal neurogenesis by irradiation leads to impairment of contextual fear conditioning [73]. Our observation that FASD-EE mice display impaired learning on a spatial pattern separation task compared to their Sac-EE controls suggests a functional correlate of the neurogenic deficit. However, we cannot rule out the possibility that this learning delay is due to other deficiencies such as impaired dentate LTP in FASD mice.

To conclude, these studies demonstrate that moderate exposure to gestational alcohol can result in resistance to the neurogenic benefits of EE in mice. The relevance of this work to clinical FASD is underscored by a recent report describing robust adult hippocampal neurogenesis in humans [74]. The implications are that some FASD individuals may display a resistance to behavioral training and EE intervention. The current studies provide the basis for testing therapeutic strategies that might restore EE-mediated plasticity in our preclinical FASD model, by targeting mechanisms important for activity-dependent integration of postmitotic neurons. These studies also provide the framework for future investigation using inducible, cell-type specific genetic manipulation of adult generated DGCs *vs.* niche regulators to restore neurogenic function in preclinical models of FASD.
